# Dual [^68^Ga]DOTATATE and [^18^F]FDG PET/CT in patients with metastatic gastroenteropancreatic neuroendocrine neoplasms: a multicentre validation of the NETPET score

**DOI:** 10.1038/s41416-022-02061-5

**Published:** 2022-11-25

**Authors:** David L. Chan, Aimee R. Hayes, Ioannis Karfis, Alice Conner, Luke Furtado O’Mahony, Magdalena Mileva, Elizabeth Bernard, Paul Roach, Gwennaëlle Marin, Nick Pavlakis, Geoffrey Schembri, Gopinath Gnanasegaran, Clementine Marin, Bruno Vanderlinden, Shaunak Navalkissoor, Martyn E. Caplin, Patrick Flamen, Christos Toumpanakis, Dale L. Bailey

**Affiliations:** 1 Medical Oncology, ENETS Centre of Excellence, Royal North Shore Hospital, Sydney, NSW Australia; 2 Faculty of Medicine and Health, University of Sydney, Sydney, NSW Australia; 3 Neuroendocrine Tumour Unit, ENETS Centre of Excellence, Royal Free Hospital, London, UK; 4 Nuclear Medicine, Institut Jules Bordet-Université Libre de Bruxelles, Brussels, Belgium; 5 University College London Medical School, University College London, London, UK; 6 Nuclear Medicine, ENETS Centre of Excellence, Royal North Shore Hospital, Sydney, NSW Australia; 7 Nuclear Medicine, Royal Free Hospital, London, UK; 8 Medical Physics, Institut Jules Bordet-Université Libre de Bruxelles, Brussels, Belgium

**Keywords:** Neuroendocrine cancer, Prognostic markers, Disease-free survival, Cancer imaging, Radionuclide imaging

## Abstract

**Background:**

Gastroenteropancreatic neuroendocrine neoplasms (GEPNENs) are heterogeneous in clinical course, biology, and outcomes. The NETPET score predicts survival by scoring uptake on dual [^68^Ga]DOTATATE and [^18^F]FDG PET/CT scans. We aimed to validate previous single-centre findings in a multicentre, international study.

**Methods:**

Dual scans were assigned a NETPET score of P1 (DOTATATE positive/FDG negative), P2–4 (DOTATATE positive/FDG positive), or P5 (DOTATATE negative/FDG positive). NETPET score, histological grade, age at diagnosis, and presence/absence of extrahepatic disease were compared to overall survival/time to progression on univariate and multivariate analysis.

**Results:**

319 metastatic/unresectable GEPNEN patients were included. The NETPET score was significantly associated with overall survival and time to progression on univariate and multivariate analysis (all *p* < 0.01). Median overall survival/time to progression was 101.8/25.5 months for P1, 46.5/16.7 months for P2–4, and 11.5/6.6 months for P5. Histological grade correlated with overall survival and time to progression on univariate and multivariate analysis (all *p* < 0.01), while presence/absence of extrahepatic disease did not. Age at diagnosis correlated with overall survival on univariate and multivariate analysis (*p* < 0.01). The NETPET score also correlated with histological grade (*p* < 0.001).

**Conclusion:**

This study validates the NETPET score as a prognostic biomarker in metastatic GEPNENs, capturing the complexity of dual PET imaging.

## Background

Neuroendocrine neoplasms (NENs) are uncommon, heterogeneous cancers which are increasing in incidence and are challenging to manage [1]. They arise from the diverse distribution of neuroendocrine cells throughout the body and are most commonly located in the small bowel, pancreas, and lungs. Depending on their site of origin, NENs can secrete hormones which result in a variety of symptoms, termed ‘functional’ disease. Whilst the standard of care for localised NENs is surgical resection, this is not always curative, and some may eventually recur. Additionally, many patients have already developed locally advanced or metastatic disease at the time of diagnosis.

Patients with NENs have extremely variable clinical courses, and much of this can be explained by the histological grade. For gastroenteropancreatic NENs (GEPNENs), histological grade is determined by the assessment of morphology, mitotic rate and proliferation (Ki-67) index [2]. Low-grade tumours may have a very indolent course and are sometimes managed by observation alone when not associated with functional symptoms. For patients with intermediate grade or progressive disease, treatments include biological agents (such as somatostatin analogues [SSA]), molecular targeted agents (such as everolimus), and peptide receptor radionuclide therapy (PRRT). In contrast, high-grade tumours, especially poorly differentiated neuroendocrine carcinomas, are generally treated with chemotherapy, and portend a poor prognosis regardless of treatment choice.

Accurate determination of grade in NENs is hampered by several potential barriers. Firstly, the common primary sites for NENs may be difficult to biopsy. The consequent choice of a fine needle biopsy may yield a small number of NEN cells which are inadequate for accurate measurement of the Ki-67 index [3]. Secondly, different sites of disease in the same patient may have different grades (intra-patient tumoural heterogeneity) and respond differently to therapies. However, sampling each site of metastatic disease is impractical and unsafe in many cases. Finally, histological grade may evolve in the same patient over time, (for example, from a grade 1 neuroendocrine tumour (NET) to a grade 3 well-differentiated NET), which predicts a poorer prognosis [4].

Positron Emission Tomography/Computed Tomography (PET/CT) scans have great applicability in NENs to predict biology and guide optimal therapeutic choices, as well as help to provide accurate prognosis. [^18^F]fluorodeoxyglucose (FDG) PET uptake corresponds to glycolytic activity in the tumour and predicts an aggressive disease course, higher histological grade, and poorer prognosis regardless of grade [5, 6]. In contrast, [^68^Ga]DOTA-SSTR (TATE/TOC/NOC) PET targets the somatostatin receptor (SSTR), especially subtype 2. Increased uptake on [^68^Ga]DOTA-SSTR PET reflects receptor density and correlates with well-differentiated histology, favourable outcomes [7] and better response to PRRT [8, 9]. The combination of imaging metabolism ([^18^F]FDG PET) and receptor (target) expression ([^68^Ga]DOTA-SSTR PET) – referred to as “dual PET imaging” – provides a comprehensive overview of the status of the disease throughout the entire body.

However, reporting dual scan findings, especially in a text-based report, results in increasingly complex descriptions of disease status at different anatomical sites. To address this, our group previously proposed the NETPET score, a 5-point scoring system for dual PET reporting in subjects with metastatic NENs, that summarises the information provided in [^68^Ga]DOTA-SSTR/[^18^F]FDG PET scans into a single parameter [10]. This score correlated with overall survival in a small sample of 62 patients with NENs of various primaries [10]. However, the single-centre nature of the study and the variety of primary sites of disease included limited the confident translation of the results into clinical practice. The current multicentre, international, retrospective study aimed to investigate whether the NETPET score retained its prognostic power in a large group of patients with metastatic GEPNENs.

## Methods

### Subject cohort

Adult subjects with histologically confirmed, metastatic GEPNEN who underwent [^68^Ga]DOTATATE PET/CT and [^18^F]FDG PET/CT at the Royal North Shore Hospital (Australia), Royal Free Hospital (UK) and Institut Jules Bordet (Belgium) were identified. Subjects were included if the scans occurred within 90 days of each other, with the first such available pair in each patient being used for analysis. A follow-up period of at least 30 days was required.

The co-primary endpoints of this study were overall survival (OS) and time to progression (TTP).

### Imaging

Image data were acquired on comparable current generation PET/CT scanners at each centre with Time-of-Flight capabilities as whole-body scans (vertex of skull to mid-thigh). [^68^Ga]DOTATATE PET/CT images were acquired between 45 and 70 min following injection of ~100–200 MBq of [^68^Ga]DOTA‐(Tyr^3^)‐octreotate, with 6–10 bed positions of 150–250 s/bed. [18 F]FDG PET/CT images were acquired between 50–60 min following injection of 250–370 MBq of [^18^F]FDG, with 6–10 bed positions of 150–240 s/bed. The exact methodology and PET image reconstruction protocol for each centre is seen in the Appendix (online only supplementary material).

### Criteria for reporting

**Fig. 1:**
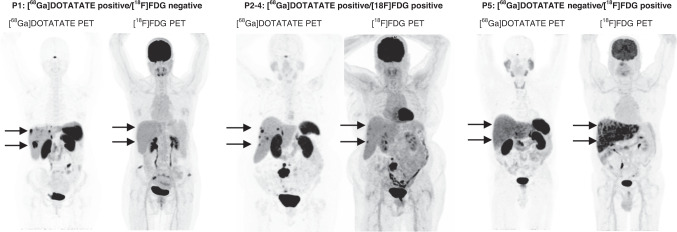
Example scan pairs representing each NETPET score category. The NETPET score was categorised into three cohorts, representing [^68^Ga]DOTATATE positive/[^18^F]FDG negative disease (P1), [^68^Ga]DOTATATE positive/[^18^F]FDG positive disease (P2–4), and [^68^Ga]DOTATATE negative/[^18^F]FDG positive disease (P5).

Subjects (*n* = 319) were assigned a NETPET score of 1–5 (as per the initial NETPET score proposal) [10] based on visual interpretation of dual PET imaging by an experienced nuclear medicine physician. The NETPET score was then categorised into three cohorts (P1, P2–4, and P5), representing [^68^Ga]DOTATATE positive/[^18^F]FDG negative disease, [^68^Ga]DOTATATE positive/[^18^F]FDG positive disease, and [^68^Ga]DOTATATE negative/[^18^F]FDG positive disease respectively, as seen in Fig. 1. Relevant clinical history from the time of scanning was provided without disclosing subsequent scan findings or clinical outcomes. Scan pairs were displayed on a dedicated nuclear medicine reporting workstation, in transverse, coronal and sagittal planes with rotating maximum intensity projection ciné images of the PET data. Dual PET scans were anatomically co-registered and locked to move in synchrony. Scans were initially windowed as per clinical practice, with [^68^Ga]DOTATATE PET/CT typically displayed at SUV 0–15 and [^18^F]FDG PET/CT at SUV 0–7. Each NETPET scoring physician had full access to the software tools used in clinical practice, including window/level adjustment, alternative colour tables, SUV regions of interest, and distance-defining callipers. Positivity on each scan was defined as uptake greater than background uptake for the specific tissue.

### Clinicopathological variables

Relevant clinicopathological variables were collected from each centre by retrospective chart review. These included age at diagnosis, gender, primary site of tumour, histological grade of tumour by WHO 2019 criteria [2], Ki-67 index, mitotic count, presence/absence of extrahepatic disease, and functionality.

OS was calculated from the time of scanning (the date of the latter scan of the pair) to the date of death and censored at the last known follow-up. TTP was similarly calculated from the time of scanning to the date of progression, as judged by the multidisciplinary team at each ENETS Centre of Excellence by review of radiological and functional imaging. Patients without progression were censored at the date of last imaging assessment.

### Statistical analysis

Covariates assessed for prognostic relevance in this cohort were age at diagnosis, histological grade, presence/absence of extrahepatic disease, and NETPET score. The variables were analysed as categorical, except for age which was analysed as a continuous variable. Unknown values were excluded from relevant analyses.

The Kaplan–Meier method was used to construct survival curves for OS and TTP. Univariate analysis was performed using the log-rank test (for categorical variables) or univariate Cox regression (for continuous variables) to assess the association between OS/TTP and the covariates listed above. Additional between-group comparisons were made for NETPET score and for grade using the log-rank test with Bonferroni correction. We also investigated the relationship between histological grade and the NETPET score using Kendall’s Tau test. Multivariate analysis was performed for OS/TTP with all covariates listed above using the Cox proportional hazard model. The assumption of proportionality was checked using Schoenfeld residuals. Covariates were also assessed for collinearity using multivariate linear regression. The distribution of clinicopathological characteristics between participating centres was investigated using the Chi-squared test. Cohen’s kappa was used to measure blinded inter-rater and intra-rater scoring reliability using a random sample of 45 patients, distributed equally over initial NETPET score and centre.

Between-group analyses were performed using GraphPad Prism 9. Schoenfeld residuals were checked using R software version 3.6.1. All other statistics were performed using SPSS 28.0.

## Results

Eligible patients (*n* = 319) were identified across the three participating centres of Royal North Shore Hospital (*n* = 151), Royal Free Hospital (*n* = 86), and Institut Jules Bordet (*n* = 82). The mean age at diagnosis was 58 years (range 22–86), and 49% of subjects were male. Dual scans were a maximum of 90 days apart, with a median scan interval of 9 days. Midgut NENs were the most common primary (52%), followed by pancreatic (36%), hindgut/rectum (7%), and other (5%). The median mitotic count was 1 per 2 mm^2^, and the median Ki-67 index was 5%. Using the WHO 2019 histological grading system [2], 29% of subjects were grade 1, 51% were grade 2, 15% were grade 3, and 5% had an unknown grade. Fourty percent of patients had functional tumours. Patient characteristics are summarised in Table 1. The distribution of primary site, histological grade, and NETPET score did not significantly differ between the three centres. The cohort median OS was 52.1 months, and the cohort median TTP was 17.1 months. The median OS follow-up time of surviving patients was 28 months.

**Table 1:** Cohort summary characteristics.

Characteristics	Subgroup	Subjects (*n* = 319)	Subjects (%)
Age at diagnosis	Mean (SD)	314	58 (12.4)
	Range		22–86
Gender	Male	156	49%
	Female	163	51%
Primary site of tumour	Midgut	167	52%
	Pancreas	114	36%
	Hindgut/rectum	23	7%
	Other	15	5%
Histological grade	Grade 1	91	29%
	Grade 2	162	51%
	Grade 3 (differentiation)	49	15% (8% WD, 6% PD, 1% unknown)
	Unknown grade	16	5%
NETPET score	P1	88	28%
	P2–4	193	61%
	P5	38	12%
Extrahepatic disease	Yes	264	83%
	No	55	17%
Functional disease	Yes	127	40%
	No	160	50%
	Unknown	32	10%

*WD* well-differentiated, *PD* poorly differentiated.

**Fig. 2:**
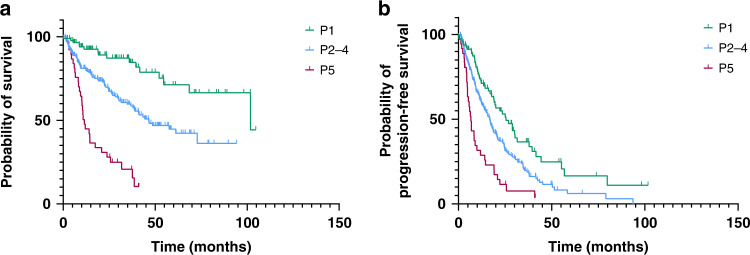
Kaplan–Meier curves for overall survival and time to progression of subjects grouped by NETPET score. Overall survival (**a**) of P1 (*n* = 88) was 101.8 months, P2–4 (*n* = 193) was 46.5 months and P5 (*n* = 38) was 11.5 months, *p* < 0.001 log-rank test. Time to progression (**b**) of P1 was 25.5 months, P2–4 was 16.7 months and P5 was 6.6 months, *p* < 0.001 log-rank test.

Using the NETPET scoring system, 28% of subjects were P1, 61% were P2–4, and 12% were P5. The median OS by NETPET score was 101.8 months for P1, 46.5 months for P2–4, and 11.5 months for P5 (*p* < 0.001, Fig. 2A). The median TTP by NETPET score was 25.5 months for P1, 16.7 months for P2–4, and 6.6 months for P5 (*p* < 0.001, Fig. 2B). The overall survival for grade 2 and 3 subgrouped by NETPET score was significantly different (Supplementary Fig. S1B, C, *p* < 0.002). Exploratory multiple comparison tests were run with Bonferroni correction to compare the OS curves of NETPET scores, and of histological grade. NETPET score comparisons were statistically significant, with *p* < 0.001 for P1 vs P2–4, P1 vs P5, and for P2–4 vs P5. Histological grade comparisons were also statistically significant, with *p* = 0.015 for grade 1 vs 2, *p* < 0.001 for grade 1 vs 3, and *p* < 0.001 for grade 2 vs 3.

On univariate analysis, OS was significantly associated with age at diagnosis (*p* = 0.003), histological grade (*p* < 0.001), and differentiation of grade 3 NENs (*p* < 0.01, Supplementary Fig. S2), but not the presence/absence of extrahepatic disease (*p* = 0.101, Table 2). TTP was significantly associated with histological grade (*p* < 0.001), but not age at diagnosis (*p* = 0.440) nor presence/absence of extrahepatic disease (*p* = 0.559, Table 3).

**Table 2:** Results of univariate and multivariate analysis for overall survival.

Variable		Univariate analysis	Multivariate analysis
NETPET score	Overall	*p* < 0.001	*p* < 0.001 HR = 2.376 (95% CI = 1.682–3.357)
	P1 vs P5	*p* < 0.001 HR = 0.375 (95% CI = 0.244–0.573)	*N/A*
	P2–4 vs P5	*p* < 0.001 HR = 0.337 (95% CI = 0.186–0.609)	*N/A*
	P1 vs P2–4	*p* < 0.001 HR = 0.133 (95% CI = 0.065–0.274)	*N/A*
Histological grade	Overall	*p* < 0.001	*p* = 0.002 HR = 1.615 (95% CI = 1.192–2.187)
	Grade 1 vs grade 2	*p* = 0.0145 HR = 0.564 (95% CI = 0.366–0.8695)	*N/A*
	Grade 2 vs grade 3	*p* < 0.001 HR = 0.423 (95% CI = 0.2498–0.7169)	*N/A*
	Grade 1 vs grade 3	*p* < 0.001 HR = 0.258 (95% CI = 0.1415–0.4700)	*N/A*
Age at diagnosis		*p* = 0.003 HR per 10 years of age=1.243 (95% CI = 1.082–1.452)	*p* = 0.009 HR per 10 years of age=1.210 (95% CI = 1.021–1.424)
Presence of extrahepatic disease		*p* = 0.101 HR = 1.466 (95% CI = 0.964–2.230)	*p* = 0.797 HR = 1.066 (95% CI = 0.655–1.736)

*HR* hazard ratio, *CI* confidence interval.

**Table 3:** Results of univariate and multivariate analysis for time to progression.

Variable		Univariate analysis	Multivariate analysis
NETPET score	Overall	*p* < 0.001	*p* = 0.001 HR = 1.502 (95% CI = 1.177–1.917)
	P1 vs P5	*p* = 0.003 HR = 0.611 (95% CI = 0.454–0.823)	*N/A*
	P2–4 vs P5	*p* < 0.001 HR = 0.492 (95% CI = 0.302–0.802)	*N/A*
	P1 vs P2–4	*p* < 0.001 HR = 0.317 (95% CI = 0.179.559)	*N/A*
Histological grade	Overall	*p* < 0.001	*p* < 0.001 HR = 1.708 (95% CI = 1.355–2.153)
	Grade 1 vs grade 2	*p* = 0.0015 HR = 0.660 (95% CI = 0.490–0.889)	*N/A*
	Grade 2 vs grade 3	*p* < 0.001 HR = 0.423 (95% CI = 0.267–0.669)	*N/A*
	Grade 1 vs grade 3	*p* < 0.001 HR = 0.317 (95% CI = 0.192–0.525)	*N/A*
Age at diagnosis		*p* = 0.440 HR per 10 years of age=1.041 (95% CI = 0.942–1.161)	*p* = 0.163 HR per 10 years of age=1.083 (95% CI = 0.970–1.195)
Presence of extrahepatic disease		*p* = 0.559 HR = 0.908 (95% CI = 0.650–1.269)	*p* = 0.079 HR = 0.742 (95% CI = 0.532–1.035)

*HR* hazard ratio, *CI* confidence interval.

Collinearity matrices and variance inflation factors indicated no collinearity between NETPET score, histological grade, age at diagnosis, or presence/absence of extrahepatic disease.

On multivariate analysis, OS remained significantly correlated with the NETPET score (*p* < 0.001), histological grade (*p* = 0.002), and age at diagnosis (*p* = 0.009), but not presence/absence of extrahepatic disease (*p* = 0.797, Table 2). TTP also remained significantly correlated with the NETPET score (*p* = 0.001) and histological grade (*p* < 0.001), but not age at diagnosis (*p* = 0.163) nor presence/absence of extrahepatic disease (*p* = 0.079, Table 3).

The NETPET score and histological grade were significantly correlated with each other (*p* < 0.001). We note that none of the patients with a P5 classification had grade 1 histology. Conversely, only 1 patient with grade 3 disease had a P1 classification (i.e. [^18^F]FDG non-avid). Interestingly, 59% of patients with grade 1 disease had [^18^F]FDG avidity (resulting in a classification of P2–4), which is higher than previously reported rates of [^18^F]FDG avidity in the same population [5]. The distribution of the NETPET score stratified by histological grade can be seen in Table 4. Both inter-rater reliability (kappa = 0.8) and intra-rater reliability (kappa = 0.9) were high.

**Table 4:** Distribution of NETPET score by histological grade.

NETPET score	Grade 1	Grade 2	Grade 3	Grade 3 Differentiation	Total
P1	37 (41%)	43 (26%)	1 (2%)	1 WD (100%)	81 (27%)
P2–4	54 (59%)	103 (63%)	28 (57%)	20 WD (71%) 6 PD (21%) 2 unk (7%)	185 (61%)
P5	0 (0%)	17 (10%)	20 (41%)	4 WD (20%) 14 PD (70%) 2 unk (10%)	37 (12%)
Total	91	163	49		303^a^

^a^Total excluding those with unknown grade (*n* = 16). *WD* well-differentiated, *PD* poorly differentiated, *unk* unknown.

## Discussion

The current study is a multicentre international collaboration combining cohorts of patients with metastatic GEPNEN who had undergone dual PET imaging on comparable current generation PET/CT scanners and populations. GEPNEN patient data from individual centres has been reported in-part previously [10–12], but the analysis of combined multicentre, international data makes this the largest study to date to investigate the prognostic value of dual PET imaging. We conclude that the NETPET score is significantly prognostic for OS and TTP on both univariate and multivariate analyses.

The NETPET score stratifies patients with metastatic GEPNENs into three prognostic classes, which strongly predict for OS and TTP after accounting for histological grade and other known prognostic factors. The NETPET score is correlated with histological grade but may also highlight situations where historically “under-grading” has occurred (such as reliance on a biopsy from a single site for prognostication). Whilst dual PET imaging can be considered for all patients with a diagnosis of metastatic GEPNEN, it may be of particular utility for grade 2–3 GEPNENs which have wide biological heterogeneity, where the results of a single biopsy site may not reflect the overall biology of the disease in individual patients.

Previous studies from the three participating centres have investigated the prognostic value of the NETPET score in smaller groups of patients [10, 11, 13, 14], with Chan et al. [10] including patients with primaries of any site. Similar smaller studies have also been conducted in bronchial NENs; and whilst the prognostic impact of the NETPET score was preserved [15], a significant minority of these patients may be non-avid on both [^68^Ga]DOTATATE and [^18^F]FDG PET, a finding that deserves further investigation [16]. Dual PET imaging has also been investigated in other settings, such as in directing management of GEPNENs [17] and in diagnostic NEN workup [18], and prior to PRRT [19]. The current study performed a repeat interrogation of relevant databases to identify eligible patients imaged since the completion of the original studies. The current study is one of the few to investigate the presence/absence of both [^68^Ga]DOTATATE and [^18^F]FDG avidity in the same tumour using spatial correlation, rather than just determining overall avidity on each scan individually. A significant correlation was noted in this study between histological grade and the NETPET score. A patient with P5 classification in the current cohort was extremely unlikely to have low-grade disease; additionally, a patient with grade 1 disease was unlikely to have a P5 classification (i.e. [^18^F]FDG avid, [^68^Ga]DOTATATE non-avid disease).

The strengths of this study include the large number of patients with an uncommon disease from three independent centres in an international collaboration, and the use of harmonised acquisition protocols in all three PET systems. The same objective and reproducible imaging analysis protocol was also applied across all centres by nuclear medicine physicians with expertise in NENs, with high inter-rater and intra-rater reliability. A large number of potential prognostic covariates were collected and allowed for multivariate analysis, which is important in a heterogeneous disease entity such as NENs. We acknowledge that the retrospective nature of this study makes it susceptible to bias, although consecutive series of patients were identified at each participating centre. Furthermore, the interval between PET scans (maximum 90 days) allows the potential for lesions to shift or grow, impeding accurate scan comparison. However, this is unlikely to significantly impact our data given the median interval between the two scans was 9 days, while the median cohort TTP was 17.1 months. We have not reported the impact of different treatments received by individual patients, potentially introducing bias in the TTP and OS analysis. Particularly, the commencement of PRRT may present a survival advantage in the P1–4 cohort, which is currently being investigated by the authors. We also acknowledge the non-standardised approach to determine progression as a limitation of this study. The above findings should ideally be confirmed in a prospective clinical trial, using the NETPET score as an exploratory/novel endpoint, although the uncommon nature of NENs would make such a study difficult to accrue.

Whilst the current study is a retrospective analysis, several changes to clinical practice in NENs should be considered as a result. The use of the NETPET score as a prognostic biomarker is supported by its significance on univariate and multivariate analysis, and it may serve to identify patients with a potentially aggressive disease course. Furthermore, the utility of dual PET imaging is highlighted by 59% of grade 1 patients (a group typically seen as ‘low risk’) demonstrating [^18^F]FDG avidity. These patients may be considered for closer follow-up or more aggressive choices of systemic treatment, perhaps pending a dual PET guided biopsy to identify potential development of high-grade disease (i.e. the site of greatest [^18^F]FDG avidity). Importantly, this study highlights the potential contribution of dual PET imaging in directing the care of patients with advanced NENs. Further research is needed to determine if the NETPET score can also predict response to treatment on repeat imaging.

Histological grade and differentiation are well-established markers for prognosis, and remain so in our overall cohort (Supplementary Fig. S2). Preliminary results (Supplementary Fig. S1) suggest that the NETPET score further stratifies traditional histological grading, however, larger numbers and longer follow-up is required to validate this small subgroup analysis. Similarly, accrual of larger numbers of grade 3 patients is required to assess whether the NETPET score is prognostic within the well-differentiated and poorly differentiated subgroups. This is important for future evaluation as the NETPET score potentially highlights areas of more aggressive disease for monitoring and/or additional treatment (such as radiotherapy), where traditional histology does not.

We recommend dual PET imaging for patients with rapidly progressing disease (irrespective of original biopsy findings), and those with grade 2 or well-differentiated grade 3 disease, in order to identify areas of potentially [^18^F]FDG avid, non-[^68^Ga]DOTATATE avid (i.e. discordant) disease. Such sites may not respond to SSTR-dependent therapies, thus influencing treatment selection. However, we acknowledge that economic considerations such as funding for dual tracers may limit the widespread adoption of routine dual PET imaging into clinical practice.

Several avenues of exploration are suggested by the findings presented here. Quantitative analysis of both [^18^F]FDG and [^68^Ga]DOTATATE PET scans is underway in the current cohort, in order to compare the prognostic value of PET-volumetric data based on each scan, an area previously investigated in smaller cohorts [10, 20, 21]. Lesion-based analysis and comparison of histopathological characteristics to PET findings may provide unequivocal evidence that PET imaging can act as a “virtual biopsy” to predict disease biology and evolution. It remains unknown whether the NETPET score may predict for the efficacy of PRRT, chemotherapy, and other systemic treatments such as molecular targeted therapy and SSAs. Therefore, validation of the NETPET score to predict treatment response and inform clinical management is of high interest. Finally, translational research into the cellular and molecular underpinnings of each [^18^F]FDG/[^68^Ga]DOTATATE phenotype will enable further insight into the biological behaviour predicted by dual PET imaging, ultimately leading to better therapy selection and superior patient outcomes.

Our large multicentre study validates the NETPET score as a robust prognostic biomarker of OS and TTP in patients with metastatic GEPNEN, and it represents a valuable complement to the prognostic algorithm. Dual PET imaging should be considered in all patients with a diagnosis of metastatic GEPNEN to guide the most optimal site for biopsy and inform the management approach.

## Supplementary information

Supplementary Figure 1

Supplementary Figure 2

Supplementary Figure Legends

Appendix

Reproducibility Checklist

## Data Availability

The datasets analysed during the current study are not publicly available due privacy and ethical restrictions, but are available from the corresponding author on reasonable request and relevant ethics approval.
