# Supplementary figures and images for: Dual [68Ga]DOTATATE and [18F]FDG PET/CT in patients with metastatic gastroenteropancreatic neuroendocrine neoplasms: a multicentre validation of the NETPET score
Source: Br J Cancer. 2022 Nov 25;128(4):549–55. doi: 10.1038/s41416-022-02061-5 (PMC9938218; doi:10.1038/s41416-022-02061-5)

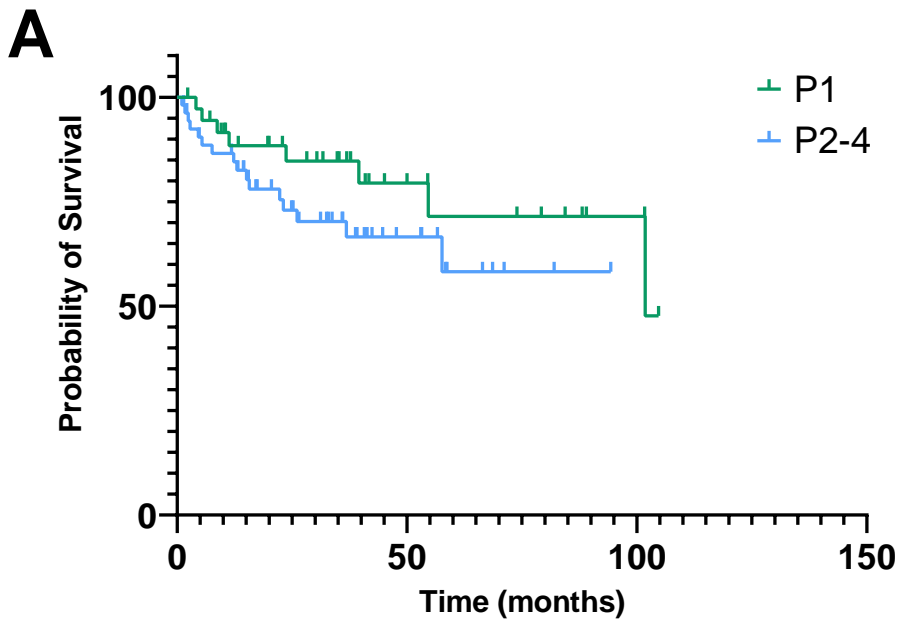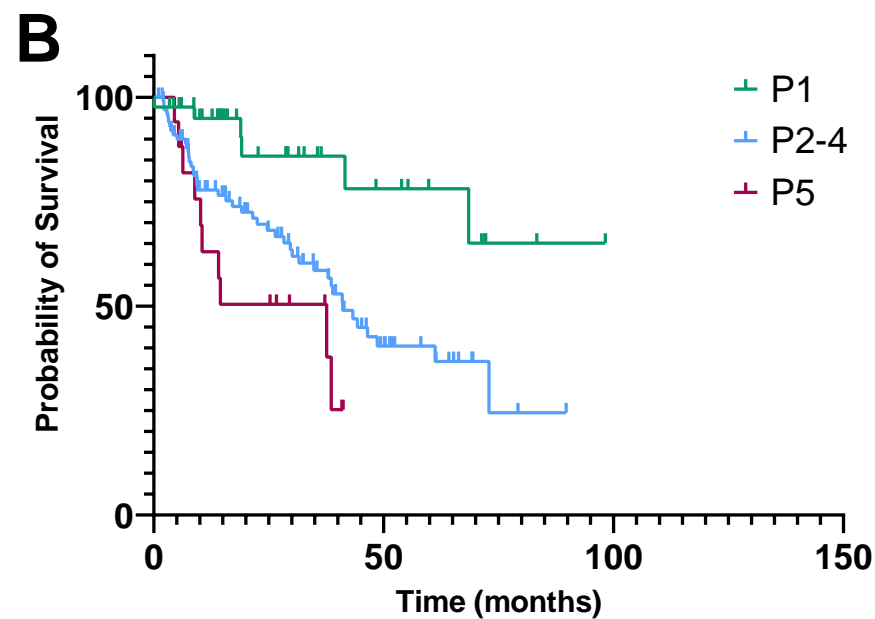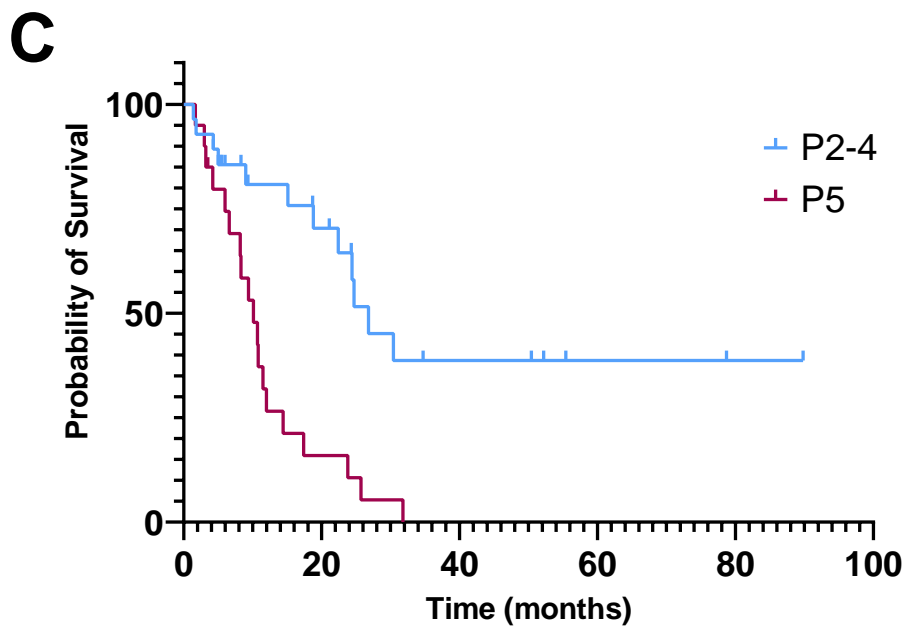

Supplement: Supplementary file 1 — Supplementary Figure 1 [file 41416_2022_2061_MOESM1_ESM.pdf]

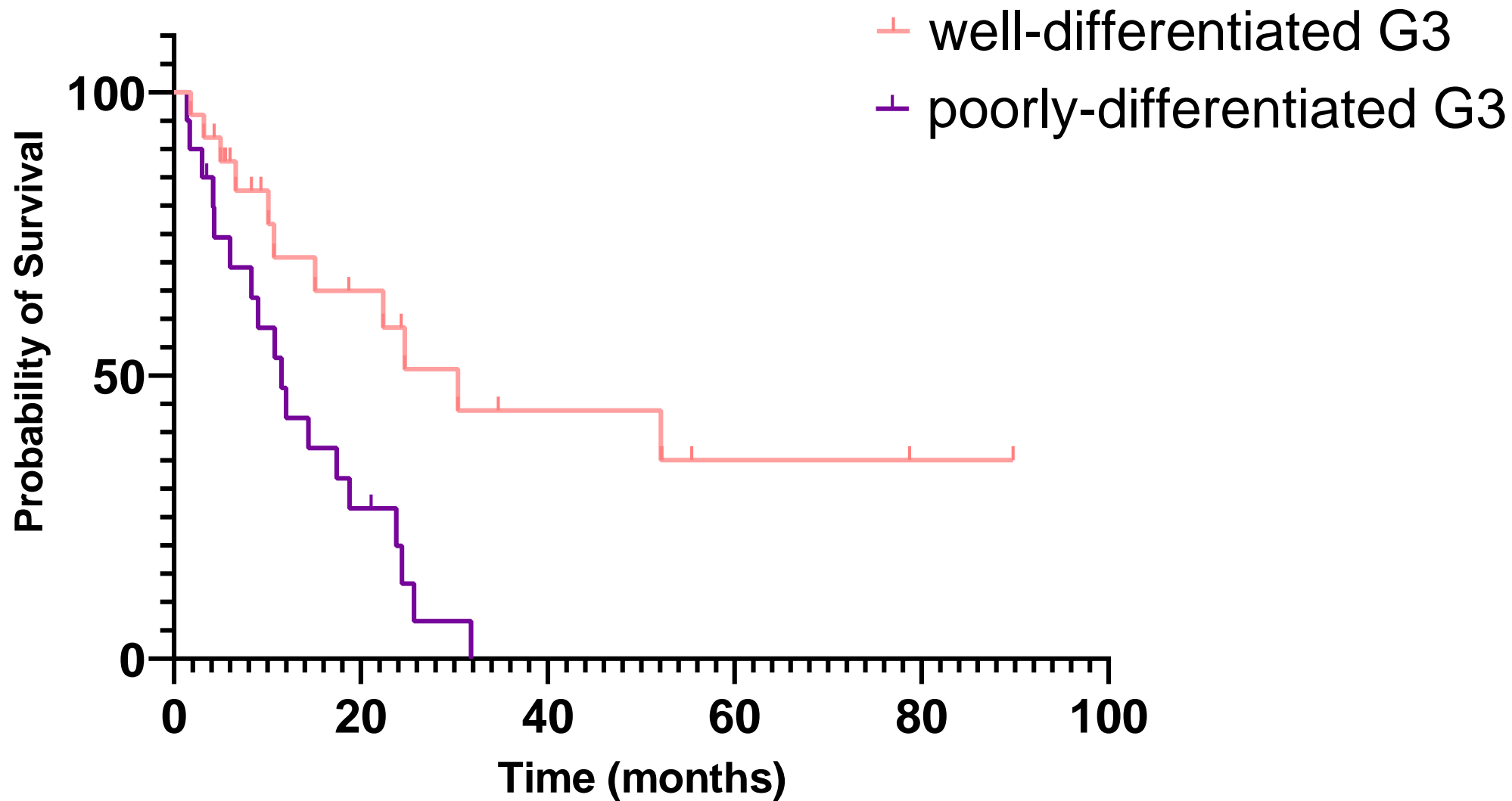

Supplement: Supplementary file 2 — Supplementary Figure 2 [file 41416_2022_2061_MOESM2_ESM.pdf]
