# Supplementary material for: Dual [68Ga]DOTATATE and [18F]FDG PET/CT in patients with metastatic gastroenteropancreatic neuroendocrine neoplasms: a multicentre validation of the NETPET score
Source: Br J Cancer. 2022 Nov 25;128(4):549–55. doi: 10.1038/s41416-022-02061-5 (PMC9938218; doi:10.1038/s41416-022-02061-5)
Supplement: Supplementary file 3 — Supplementary Figure Legends [file 41416_2022_2061_MOESM3_ESM.docx]

**Supplementary Figure Legends**

**Supplementary Figure 1**. Kaplan-Meier curve for overall survival of grade 1 **(A)**, grade 2 **(B)**, and grade 3 **(C)** subgroups, stratified by NETPET score. For grade 1 patients **(A)**, overall survival of P1 (*n*=37) was 101.8 months, and P2-4 (*n*=54) was not reached, *p*>0.5 log-rank test. For grade 2 patients **(B)**, overall survival of P1 (*n*=43) was not reached, P2-4 (*n*=103) was 41.1 months, and P5 (*n*=17) was 37.6 months, *p*<0.002 log-rank test. For grade 3 patients **(C)**, overall survival of P2-4 (*n*=28) was 26.8 months and P5 (*n*=20) was 10.1 months, *p*<0.001 log-rank test. The grade 3 patient scored as P1 was excluded from analysis due to the small sample size (*n*=1).

**Supplementary Figure 2.** Kaplan-Meier curve for overall survival of grade 3 (G3) subjects grouped by histological differentiation. Overall survival of G3 well-differentiated subjects (*n*=25) was 30.4 months, and G3 poorly differentiated subjects (*n*=20) was 11.5 months, *p*<0.01 log-rank test.
