# Supplementary material for: Dual [68Ga]DOTATATE and [18F]FDG PET/CT in patients with metastatic gastroenteropancreatic neuroendocrine neoplasms: a multicentre validation of the NETPET score
Source: Br J Cancer. 2022 Nov 25;128(4):549–55. doi: 10.1038/s41416-022-02061-5 (PMC9938218; doi:10.1038/s41416-022-02061-5)
Supplement: Supplementary file 4 — Appendix [file 41416_2022_2061_MOESM4_ESM.docx]

**Appendix**

**Royal North Shore Hospital (Australia) Imaging Methodology**

All image data were acquired on a PET/CT with Time-of-Flight (ToF) capabilities and 21.6cm axial field of view (Biograph mCT.S/64 PET/CT, Siemens Healthcare, Hoffman Estates, USA). Data were typically acquired as whole-body scans (top of skull to mid-thigh), usually requiring 6-8 bed positions in step-and-shoot mode.

For [^68^Ga]DOTATATE PET/CT scans, subjects were injected with 120-180 MBq of [^68^Ga]DOTA‐(Tyr^3^)‐octreotate produced in-house [21] with imaging commencing approximately 50 mins after injection with whole-body low-dose CT followed by the PET acquisition of 180 s/bed. The subjects were advised to cease all somatostatin analogues four weeks prior to the scan.

For the [^18^F]FDG scans, subjects were required to fast for at least 6 h prior to the scan and blood glucose levels were checked to ensure they were in the range 4-11 mmol/L. Subjects were administered a standard amount of 250 MBq of [^18^F]FDG if their weight was ≤ 90 kg or 300 MBq if > 90 kg. At approximately 50 mins after injection, scanning commenced with whole body low-dose CT followed by the PET acquisition of 150 s/bed.

All PET reconstructions were performed using 3D OSEM with 2 iterations and 21 subsets, applied in conjunction with a matched Gaussian post-reconstruction filter of 5mm FWHM. Reconstruction included a depth-dependent spatial resolution recovery algorithm (“TrueX”, Siemens Healthcare, Hoffman Estates, USA) in addition to standard corrections for random coincidences, scattered photons, and attenuation.

**Institut Jules Bordet (Belgium) Imaging Methodology**

All [^18^F]FDG and [^68^Ga]DOTATATE PET/CT images were acquired at the Nuclear Medicine Department of Jules Bordet Institute, as in Karfis et al. [12], using a General Electric (GE-Healthcare) Discovery 690 ToF PET system. Before [^18^F]FDG injection, patients fasted for at least 6 h and had blood glucose levels below 150 mg/dL. Long-acting somatostatin analogues were discontinued at least 4 weeks prior to [^68^Ga]DOTATATE PET/CT acquisition. Whole-body PET images were acquired 60 mins after injection of 3.8 MBq/kg of [^18^F]FDG with 8 bed positions of 90 s with an overlap of 23%, and 60 min after injection of 2MBq/kg of [^68^Ga]DOTATATE with 10 bed positions of 150–180 s with an overlap of 23%. PET images were reconstructed with GE built-in algorithms; VUE Point FX for [^18^F]FDG Ordered Subset Expectation Maximization (OSEM) algorithm with 2 iterations and 18 subsets, 6.4 mm Full-Width at Half-Maximum (FWHM) Gaussian post-reconstruction filtering, and TOF attenuation and scatter corrections) and VUE Point FXS for [^68^Ga]DOTATATE (OSEM algorithm with 3 iterations and 18 subsets, 6.8 mm FWHM Gaussian post-reconstruction filtering, and ToF, attenuation, scatter, and resolution recovery corrections).

**Royal Free Hospital (UK) Imaging Methodology**

As in Hayes et al. [11] the injected activity was approximately 150 MBq of [^68^Ga]DOTATATE (range 100-200 MBq). Images were acquired 45–70 min after radiotracer administration. The mass quantity of [^68^Ga]DOTATATE peptide that the patients received was in the range of 10–40 μg. Radiolabelling was performed in-house via radiopharmacy using the synthesis method with PharmTracer Cassette and Reagents from Eckert and Ziegler (Berlin, Germany). No prior preparation was required for tracer injection i.e. patients on long-acting somatostatin analogues did not stop their injection. Imaging was performed using dedicated ToF PET/CT cameras (Siemens Biograph mCT.S/64) combining PET with a 64-slice CT. Whole-body imaging was performed from the vertex to mid-thighs with the patient supine. PET images were acquired for 4 min per bed position. PET images were reconstructed using an ordered-subsets expectation maximization algorithm with 2 iterations and 21 subsets and with CT-based scatter and attenuation correction. [^18^F]FDG PET/CT was acquired at approximately 60 min after radiotracer administration with activity of 3 MBq per kilogram of body weight up to a maximum of 370 MBq. Images were acquired for 3-4 min per bed position (depending on body mass index). PET images were reconstructed using an ordered-subsets expectation maximization algorithm with 2 iterations and 21 subsets and with CT-based scatter and attenuation correction.
